# The Effects of Melatonin on the Physical Properties of Bones and Egg Shells in the Laying Hen

**DOI:** 10.1371/journal.pone.0055663

**Published:** 2013-02-28

**Authors:** Alexander C. Taylor, Maria Horvat-Gordon, Ashli Moore, Paul A. Bartell

**Affiliations:** 1 Department of Animal Science, The Pennsylvania State University, University Park, Pennsylvania, United States of America; 2 Intercollege Graduate Degree Program in Physiology, The Pennsylvania State University, University Park, Pennsylvania, United States of America; 3 Department of Biology, University of Virginia, Charlottesville, Virginia, United States of America; Morehouse School of Medicine, United States of America

## Abstract

Laying hens often experience unbalanced calcium utilization which can cause deficiencies in bone and egg mineralization. Because melatonin has been shown to affect bone mineralization in other animals, we examined whether treating hens with melatonin would affect eggshell thickness and improve skeletal performance, thereby reducing skeletal and egg shell defects. Birds were given a diet containing either low (30 µg/kg), medium (300 µg/kg), or high (3 mg/kg) concentrations of melatonin, or control feed through approximately one laying cycle. We examined the weight, length, and strength of egg, femur, tibia, and keel. Hens treated with a high concentration of melatonin showed significant strengthening in their femur and tibia, as measured by maximum force sustained and breaking force, compared to controls. Egg weights from hens treated with melatonin were significantly greater than those from hens that were not treated with melatonin. Conversely, egg shell mass of hens treated with melatonin was significantly lower than those of hens not treated with melatonin. Our data suggest that melatonin may affect the allocation of calcium to bone at the expense of egg shell mineralization.

## Introduction

Laying hens have an average daily calcium turnover of approximately 1 g/kg body weight, giving them a proportional daily calcium requirement higher than virtually any other animal [1]. Each egg contains over 2.3 g of calcium in the form of calcium carbonate, an amount which is roughly equivalent to 10% of a hen's total calcium stores [2]. Birds utilize calcium more efficiently than most animals, but unbalanced calcium utilization still frequently occurs. These problems manifest as poor quality egg shells and skeletal defects, such as osteoporosis and fractures. Poor egg shell and skeletal quality are detriments to the meat and egg industries and raise ethical questions concerning animal health. Inability to form a fully protective egg shell is also a major cause of disease outbreak [3].

The avian pineal gland synthesizes melatonin in a rhythmic fashion, such that melatonin levels are highest at night. Melatonin is secreted by the pineal into the blood, where it circulates throughout the body in order to exert its effects on a myriad of target tissues. Chickens possess 3 melatonin receptor subtypes: MEL_1A_, MEL_1B_, and MEL_1C_ [4], [5], [6], [7]. These receptors each have different affinities for melatonin and exert varying effects upon binding melatonin. Consequently, melatonin receptor subtypes vary in density and abundance throughout the body. Mel_1A_ and Mel_1B_ receptors have been reported in the heart, retina, pineal, liver, and lung of zebra finches [8]. In the shell gland, MEL_1C_ has been identified as a positional candidate for affecting egg shell thickness [9]. White leghorns also express Mel_1A_ in the retina [4], Mel_1B_ in the retina [5], [6], Mel_1A_ and Mel_1B_ in the brain [7], and Mel_1A_, Mel_1B_, and Mel_1C_ in the ovary [5].

Ossification of bone occurs when a chondrocyte-derived Type I Collagen matrix is mineralized with a modified form of hydroxyapatite crystals by the activity of osteoblasts. The mineralized component of bone is periodically dissolved by osteoclasts and remineralized by osteoblasts in a process called bone remodeling. Balance of osteoblast and osteoclast activities are essential for maintaining bone integrity. Melatonin plays an important role in bone ossification and remodeling by promoting the production of Type I Collagen by osteoblasts and increasing osteoblastic proliferation [10]. Melatonin also promotes osteoblast differentiation [11], [12] and inhibits bone resorption by suppressing RANKL-mediated osteoclast formation and activation [13]. These pieces of evidence suggest that melatonin can contribute to bone formation and help limit the degradation of bone. Data suggest that melatonin may also be necessary for bone growth during early development. Pinealectomy in developing chicks, which abolishes circulating melatonin, induces a high incidence of scoliosis [14]. The incidence of this developmental bone disease is attenuated by melatonin administration [15], suggesting that lack of pineal melatonin is, in part, responsible for this deformity [15].

Melatonin has been shown to affect calcium absorption in the duodenum, the main site of calcium uptake in the avian digestive system. Additionally, melatonin and melatonin receptor agonists increase calcium uptake in a dose-dependent manner when given to cultures of isolated human or rat enterocytes [16]. Consequently, we examined whether exogenous melatonin given in feed could affect the mineral content of egg shells and bone in hens and also increase the strength of developing bones of laying hens.

## Methods

White leghorn chicks (W36, Hyline) were randomly divided into four groups [n = 20 birds per group] and given a diet containing either: low (30 µg/kg), medium (300 µg/kg), or high (3 mg/kg) concentrations of melatonin. A control group received normal feed with no melatonin. Concentrations were chosen based on the amount of melatonin reported to influence the circadian rhythm of chickens; 300 µg/kg achieves a plasma level of 15 nM, approximately 10 times the normal nocturnal levels in plasma [17]. Feed was mixed once per week to prevent melatonin degradation and was provided to birds *ad libitum*. Birds were raised under a 16∶8 hour light∶dark cycle and allowed to progress through approximately one laying cycle (approximately one year). Approximately half way through the laying cycle, eggs were collected for analysis. Birds were bled at 3, 9, 15, and 21 hours after lights-on to assay for melatonin levels to confirm that melatonin levels in circulation had been elevated. Melatonin was isolated from an aliquont of plasma using chloroform extraction and quantified using radioimmunoassay (RIA). RIA was performed using anti melatonin Antibody R1055 previously validated for use in chicken. Procedures were performed as described by Rollag and Niswender (Rollag and Niswender, 1976). All experiments were performed in strict accordance with and approval from The Pennsylvania State University Animal Care and Use Committee (IACUC #38421).

Birds were weighed and then killed by cervical dislocation. Keels, femurs, and tibiotarsi were removed, cleaned, and stored at 4°C. Femur and tibia length and diameter at the center of the diaphysis were measured with Vernier calipers (15-100-100, Manostat). One tibia from each bird was split with rongeurs and a stainless steel curette was used to remove all medullary bone from the bone cavity. Bone was placed in a Petri dish with de-ionized water, crushed with a stir bar, washed, centrifuged at 500 rpm for 5 min (Beckman Coulter, Allegra X-22R) and the water was decanted off. The process was repeated until the bone was clean of marrow, lipid, blood, and connective tissue. Bone was dried in an oven (Isotemp 630F, Fisher Scientific) at 100°C for 48 hours, cooled in a desiccator and weighed (1212MP, Sartorius) prior to ashing at 600°C for 6 hours in a muffle furnace (10-650-125, Fisher Scientific). Following ashing the bone was allowed to cool in a desiccator and then weighed again.

Bones were subjected to a 3-point break test using a universal materials tester (Autograph AGS-X 1kN, Shimadzu) controlled by proprietary software (Trapezium Lite X v 1.0.3, Shimadzu). The testing support consisted of an adjustable 2-point block jig, spaced at 50 mm, with a load sensor attached to a bending punch on the crosshead. The crosshead descended at 5 mm/min until a break was determined by measuring a reduction in force of ≥10%. The testing parameters collected included elastic force, chord force, break force, yield strength at 10% of breaking load, upper yield point, and elongation at 1N load. Upper yield point was determined in real time by the first reduction in force of >1% of current load. Once a bone was broken, cortical bone thickness was measured with Vernier calipers and entered into the tube materials profile in the software in order to analyze the parameters of the 3-point break. The tibia and femur were then dried and ashed as described above for medullary bone.

Keel bones were air dried but were not weighed as they contained varying amounts of attached cartilage. Keels were tested with the same 3-point break test, but were analyzed with a plate materials profile instead of a tube profile. Eggs were weighed with a balance and the egg yolk and whites were removed. Egg shells were washed, dried in an oven at approximately 100°C for 12 hours and then weighed again.

All data analyses were performed using Minitab Version 16 (Minitab, Inc.). One-way ANOVA with Tukey's post-hoc test with 95% confidence interval was used to compare dry weight of bone, ashed weight of bone, bone length, bone diameter, bone thickness, upper yield point, breaking strength, elastic force, yield strength, maximum force sustained, and elongation at 1N load.

## Results

**Figure 1:**
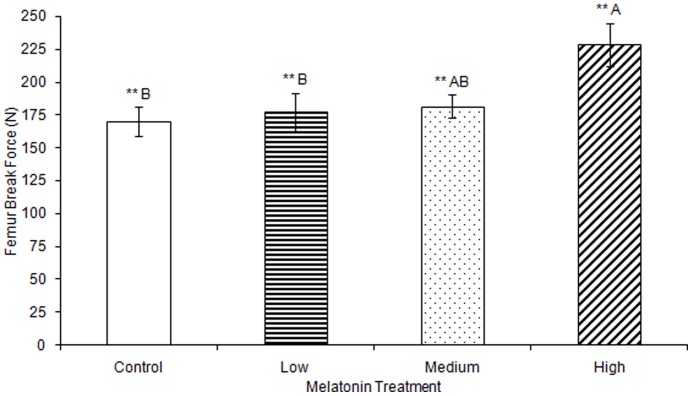
Femur break force was significantly greater in birds treated with high concentrations of melatonin (228.35±16.22) compared to birds treated with low concentrations of melatonin (177.15±14.22) or controls (170.03±11.18; p<0.01). Groups sharing a letter are not significantly different.

**Figure 2:**
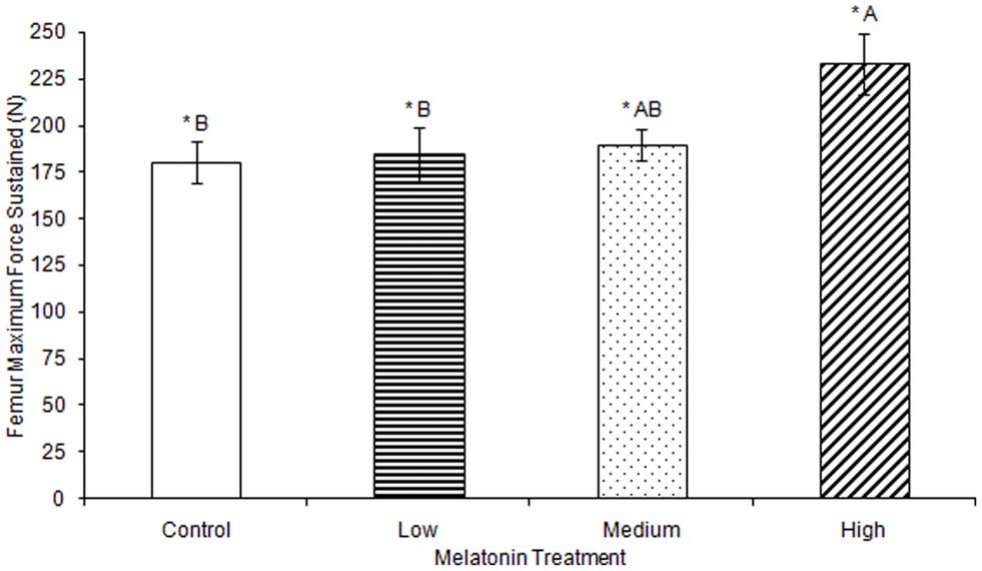
Maximum force sustained by femurs during testing was significantly greater in birds treated with high concentrations of melatonin (233.17±16.41) compared to controls (180.13±11.33) or birds treated with low concentrations of melatonin (184.55±13.97; p<0.05). Groups sharing a letter are not significantly different.

No significant differences were observed in femur ash weight between treatment groups (Table 1). Femur break force was observed to be significantly greater in birds treated with high levels of melatonin (F(3,74) = 4.25, p<0.01; Fig. 1; all results in Tables 1–3) compared to the femur break force of birds treated with low levels of melatonin or control birds. Femur break force of birds treated with high levels of melatonin was not different from those of birds treated with medium levels of melatonin. Maximum force sustained by femurs was significantly greater in birds treated with high levels of melatonin compared to birds treated with low levels of melatonin and controls (F(3,76) = 3.66, p<0.05; Fig. 2), but femurs from birds treated with medium levels of melatonin did not differ in strength from controls or from birds treated with low or high levels of melatonin.

**Table 1:** Femur Physical and Strength Properties.

Group	Length (mm)	Diameter (mm)	Thickness (mm)	Oven dry weight (g)	Ash weight (g)	Upper yield point (N)	Yield strength (N)	Break force (N)	Elastic force (N)	Max force
Control	83.95 (±3.19)	6.95 (±0.14)	0.4 (±0.03)	4.62 (±0.16)	2.63 (±0.12)	42.16 (±19.83)	65.46 (±10.53)	170.03 (±11.18)**	3985.29 (±872.37)	180.13 (±11.33)*
Low	81.39 (±0.47)	7 (±0.07)	0.43 (±0.04)	4.54 (±0.14)	2.57 (±0.14)	32.14 (±3.70)	27.23 (±2.42)	177.15 (±14.22)**	2397.54 (±578.30)	184.55 (±13.97)*
Medium	81.86 (±0.32)	7.1 (±0.07)	0.42 (±0.03)	4.54 (±0.12)	2.55 (±0.06)	47.93 (±14.71)	50.54 (±11.68)	181.73 (±8.86)**	3598.34 (±855.79)	189.81 (±8.47)*
High	81.62 (±0.39)	7.03 (±0.07)	0.48 (±0.04)	4.68 (±0.12)	2.76 (±0.12)	38.22 (±11.31)	61.98 (±19.12)	228.35 (±16.22)**	3712.61 (±1192.70)	233.17 (±16.41)*

Length, diameter, thickness, oven dry weight, ash weight, upper yield point, yield strength, break force, elastic force, and max force of tibias collected from White Leghorn hens after melatonin treatment.

* = significant at p<0.05.

** = significant at P<0.01.

*** = significant at p<0.001.

Data given are mean ± SE.

**Table 2:** Tibia Physical and Strength Properties.

Group	Length (mm)	Diameter (mm)	Thickness (mm)	Oven dry weight (g)	Ash weight (g)	Upper yield point (N)	Yield strength (N)	Break force (N)	Elastic force (N)	Max force (N)
Control	106.41 (±3.55)**	6.4 (±0.11)	0.66 (±0.06)*	5.81 (±0.19)	2.98 (±0.11)	69.74 (±15.88)	89.35 (±15.54)	173.00 (±13.21)**	5528.03 (±1183.52)	182.97 (±12.16)***
Low	114.36 (±0.87)**	6.57 (±0.12)	0.47 (±0.06)*	5.96 (±0.20)	1.88 (±1.11)	81.11 (±23.20)	75.63 (±17.97)	203.37 (±11.21)**	5317.41 (±1103.98)	225.13 (±8.86)***
Medium	115.48 (±0.51)**	6.61 (±0.12)	0.63 (±0.04)*	5.93 (±0.23)	2.88 (±0.14)	57.50 (±14.94)	91.87 (±18.41)	188.03 (±8.81)**	4179.77 (±1090.61)	207.40 (±8.26)***
High	115.55 (±0.69)**	6.37 (±0.10)	0.70 (±0.06)*	6.08 (±0.16)	3.25 (±0.12)	76.32 (±30.23)	52.84 (±12.64)	244.03 (±14.19)**	3881.62 (±768.65)	251.09 (±13.51)***

Length, diameter, thickness, oven dry weight, ash weight, upper yield point, yield strength, break force, elastic force, and max force of femurs collected from White Leghorn hens after melatonin treatment.

* = significant at p<0.05.

** = significant at P<0.01.

*** = significant at p<0.001.

Data given are mean ± SE.

**Table 3:** Egg and Shell Weights.

Group	Whole weight (g)	Shell weight (g)	Egg contents weight (g)
Control	61.67 (±0.36)***	7.90 (±0.05)***	53.77 (±0.34)***
Low	63.69 (±0.37)***	5.5079 (±0.04)***	58.18 (±0.35)***
Medium	63.58 (±0.26)***	5.4399 (±0.03)***	58.14 (±0.24)***
High	63.04 (±0.34)***	5.36 (±0.40)***	57.68 (±0.31)***

Total egg weight, shell weight, shell weight, and weight of egg contents of eggs laid by White Leghorn hens, collected after melatonin treatment.

* = significant at p<0.05.

** = significant at P<0.01.

*** = significant at p<0.001.

Data given are mean ± SE.

**Figure 3:**
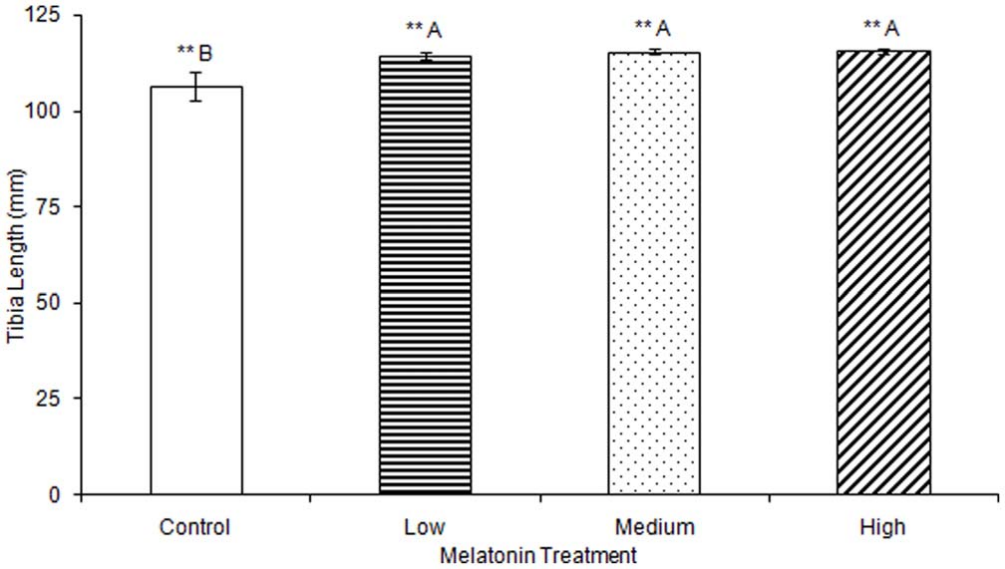
Tibias were significantly longer in birds treated with low (114.36±0.87), medium (115.48±0.51), or high (115.55±0.69) concentrations of melatonin compared to those from control birds (106.41±3.55; p<0.01). Groups sharing a letter are not significantly different.

**Figure 4:**
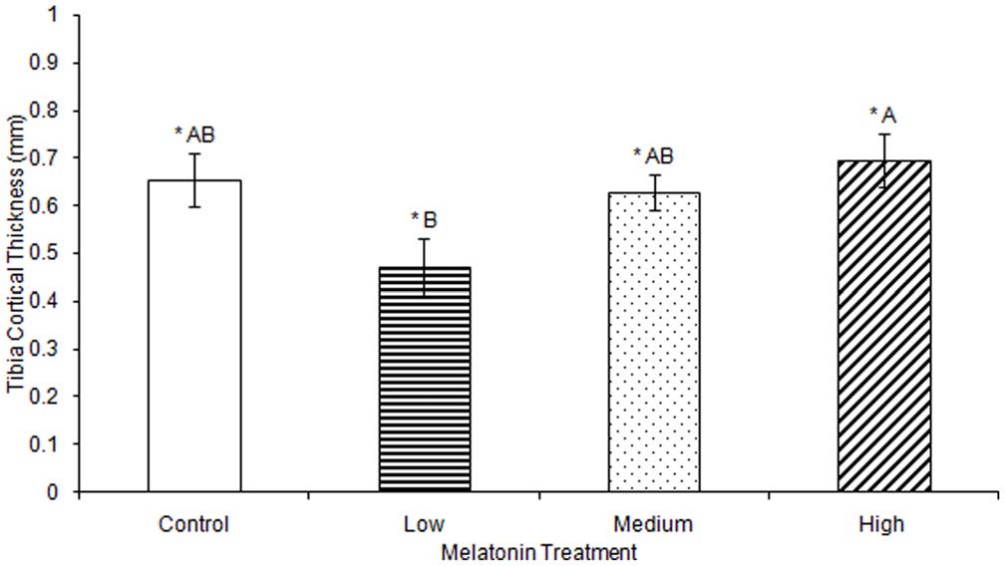
Median tibia thickness at the break was significantly greater in birds treated with high concentrations of melatonin (0.70±0.06) compared to those from birds treated with low concentrations of melatonin (0.47±0.06; p<0.05). Groups sharing a letter are not significantly different.

**Figure 5:**
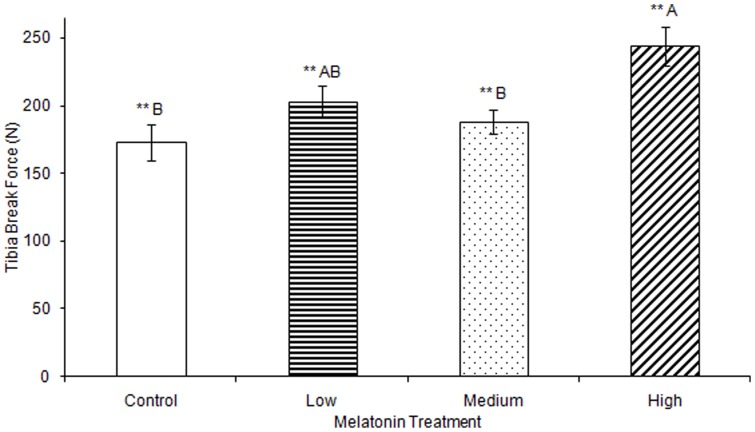
Tibia break force was significantly greater in birds treated with high concentrations of melatonin (244.03±14.19) compared to those from birds treated with low concentrations of melatonin 203.37±11.21) or those from controls (173.00±13.21; p<0.01). Groups sharing a letter are not significantly different.

**Figure 6:**
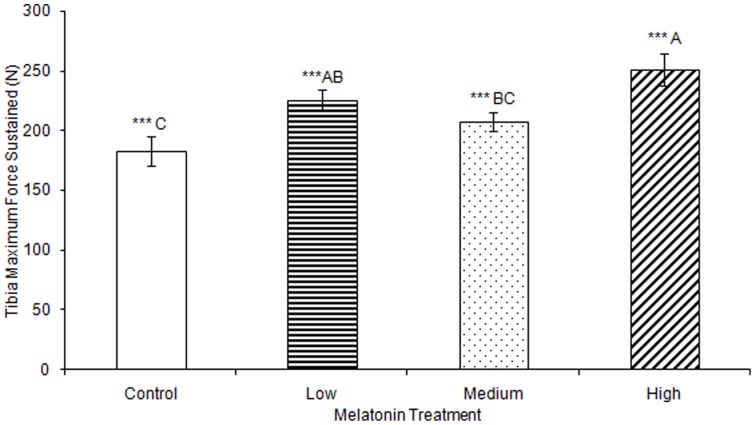
Maximum force sustained by tibias was significantly greater in birds treated with high concentrations of melatonin (251.09±13.51) compared to those from birds treated with medium concentrations of melatonin (207.40±8.26) or controls (182.97±12.16; p<0.001), and significantly greater in birds treated with low concentrations of melatonin (225.13±8.86) compared to those from control birds (p<0.001). Groups sharing a letter are not significantly different.

No significant differences were observed in tibia ash weight between treatment groups (Table 2). Tibia lengths of birds treated with high, medium, and low levels of melatonin were all significantly longer than those from controls (F(3,74) = 5.43, p<0.01; Fig. 3). Tibia cortical thickness in birds treated with high levels of melatonin was significantly greater than in birds treated with low levels of melatonin (F(3,77) = 3.29, p<0.05; Fig. 4). Tibias from birds treated with high levels of melatonin had significantly greater break force compared to controls and birds treated with low levels of melatonin, but the tibia break force of birds treated with medium levels of melatonin did not differ from birds in other treatment groups (F(3,70) = 6.67, p<0.01; Fig. 5). Maximum force sustained was significantly greater in tibias from birds treated with high levels of melatonin compared to controls or from birds treated with medium levels of melatonin, but tibia maximum force in birds treated with medium levels of melatonin did not differ compared to those from birds treated with low or high levels of melatonin (F(3,76) = 6.91, p<0.001; Fig. 6). Maximum force of tibias from birds treated with low levels of melatonin did not differ from controls.

**Figure 7:**
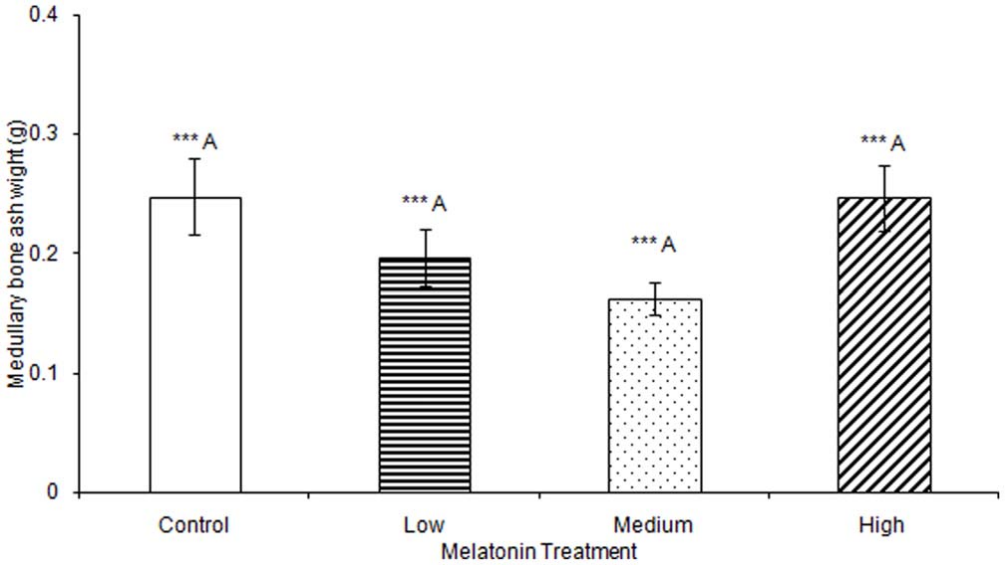
Weight of medullary bone was significantly affected by treatment (p<0.001), but no Tukey's post-hoc test pairwise comparison between controls (0.25±0.03), birds treated with low (0.20±0.01), medium, (0.16±0.00), or high (0.25±0.01) concentrations of melatonin was significant.

**Figure 8:**
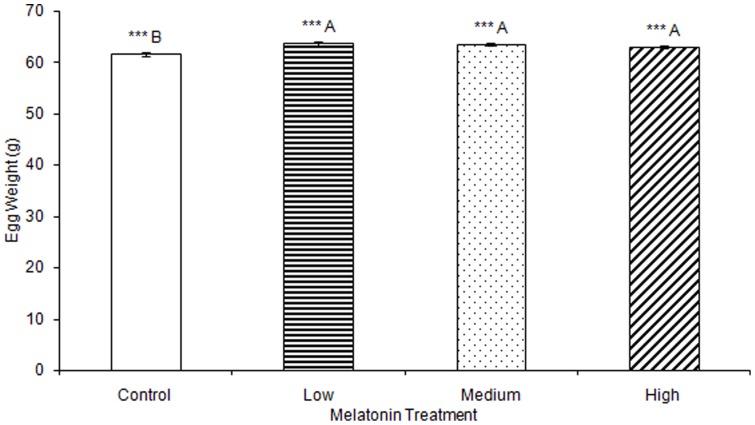
Total egg weight was significantly greater in birds treated with low (63.69±0.37), medium (63.58±0.26), or high (63.04±0.34) concentrations of melatonin compared to those laid by control birds (61.67±0.36; p<0.001). Groups sharing a letter are not significantly different.

**Figure 9:**
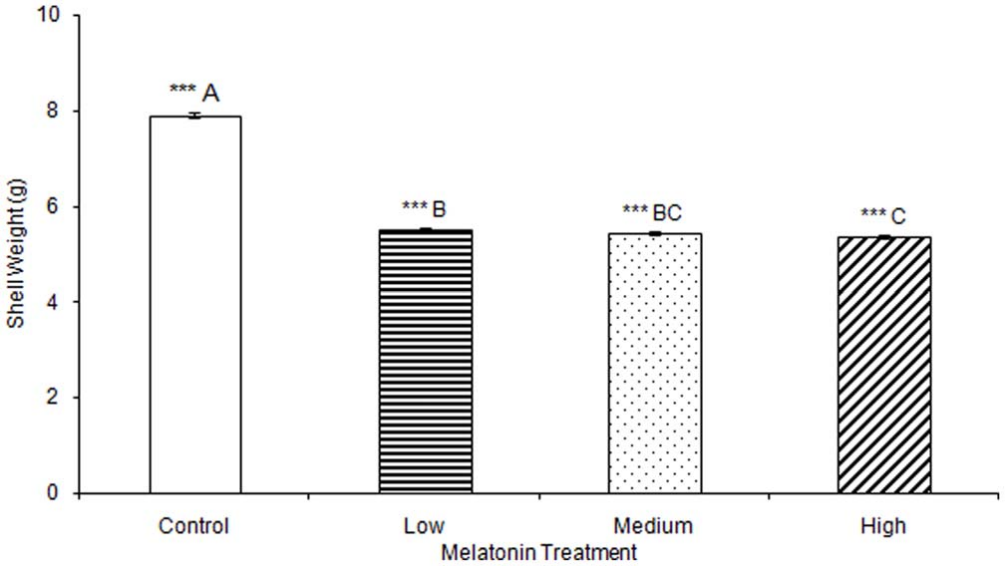
Egg shell weight was significantly greater when laid by control birds (7.90±0.05) compared to birds treated with low (5.51±0.04), medium (5.44±0.03), or high concentrations of melatonin (5.36±0.04; p<0.001). Egg shell weight was significantly greater when laid by birds treated with low concentrations of melatonin compared to eggs laid by birds treated with high concentrations of melatonin (p<0.001). Groups sharing a letter are not significantly different.

**Figure 10:**
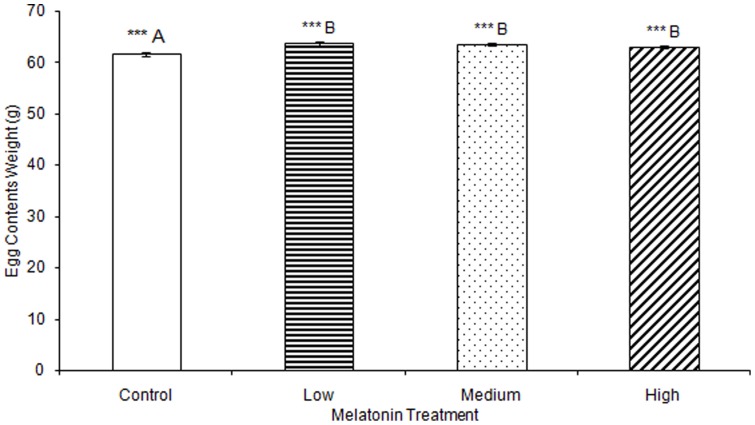
Egg content weight was significantly greater when laid by birds treated with low (58.18±0.35), medium (58.14±0.24), or high concentrations of melatonin (57.68±0.31) compared to those laid by control birds (53.77±0.34; p<0.001).

No significant differences were observed in any materials testing parameter for keels in any of the melatonin treatment groups (Table S1). One-way ANOVA determined that medullary bone ash weight was significantly affected by treatment (F(3,78) = 2.73, p<0.001), but multiple comparisons in Tukey's post-hoc test determined that there were no significant differences when comparing between birds treated with low (0.20 g±0.01), medium (0.16 g±0.00), or high (0.25 g±0.01) levels of melatonin, or controls (0.25 g±0.02; Fig. 7). Total egg weight was significantly greater in eggs laid by birds treated with low, medium, or high levels of melatonin, compared to eggs laid by control birds (F(3,805) = 6.92, p<0.001; Table 3; Fig. 8). Egg shell weight was also significantly greater in eggs laid by controls compared to those from birds treated with low, medium, or high levels of melatonin (F(3,805) = 870.89, p<0.001; Fig. 9). Egg content weight was significantly greater when laid by birds treated with low, medium, or high levels of melatonin compared to controls (F(3,805) = 41.01, p<0.001; Fig. 10).

**Figure 11:**
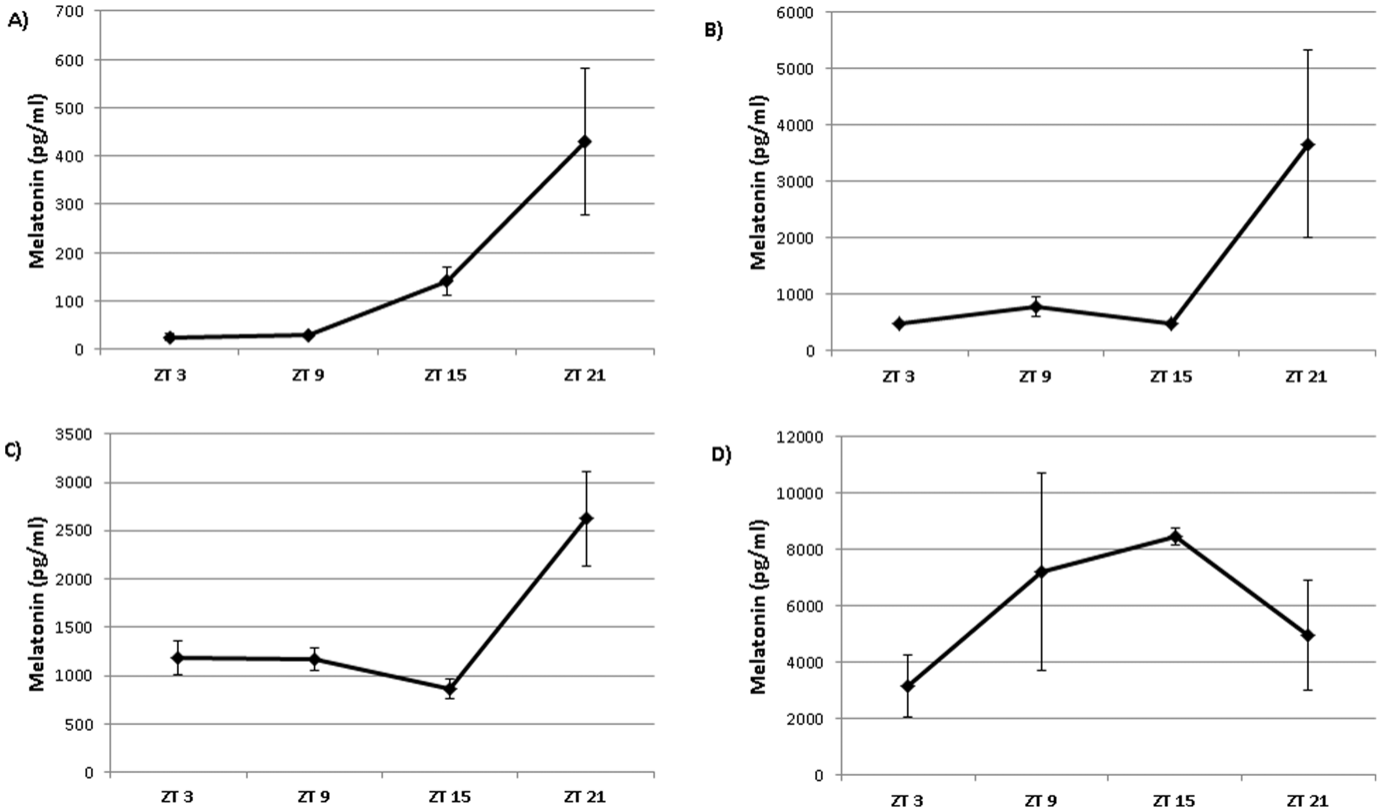
Melatonin levels in plasma from control (A), low melatonin (B), medium melatonin (C) and high melatonin (D) treated birds. Melatonin levels were rhythmic in control (p<0.02), low (p<0.02) and medium birds (p<0.05), but non-rhythmic after high melatonin treatment.

Analysis of melatonin in blood plasma showed melatonin treatment raised plasma levels of melatonin in all birds treated with melatonin (Fig. 11). Melatonin levels were raised during the daytime, but were still rhythmic across the day in low and medium melatonin treated birds (p<0.02). The levels of melatonin in birds from the high treatment group were arrhythmic, with higher levels occurring during the daytime.

## Discussion

Our work here describes the effects of dietary melatonin supplementation on bone strength. We found that melatonin increases the strength of femurs and tibias as measured by maximum force and breaking force. Melatonin has previously been shown to stimulate osteoblastic proliferation [10], [11], suggesting that melatonin could increase the rate of bone ossification. Melatonin also inhibits osteoclast formation and activation [13]. Because osteoclasts resorb bone minerals and collagen [18], melatonin supplementation could lead to increased bone mineral content by inhibiting the natural degradation of bone. Increased levels of osteoclast activity during the process of bone remodeling could lead to a more porous bone structure in a calcium-stressed bird. However, dry mass and ash weights of bones from melatonin treated birds were unchanged indicating that total organic and inorganic content in the melatonin treatment groups were not altered. These findings suggest that melatonin does not exert its strengthening effects on skeletal development by depositing additional quantities of organic matrix (e.g. collagen) or inorganic material (e.g. calcium) into the cortical bone. Our findings do not preclude an alteration of the micronutrient content in the bone matrix; the addition of trace amounts of zinc [19], [20], [21], [22] or strontium [23], [24], [25] to bone matrix can improve fracture resistance and strength without affecting overall weight or total mineral content. Melatonin has been shown to affect absorption of micronutrients, including ions [26], [27] such as zinc [28], [29].

Melatonin levels in plasma of all treated birds were higher than those of controls. In particular, plasma levels were greatly increased during the daytime, when plasma melatonin levels are normally low. It is possible that the effects of melatonin we observed on bone growth and shell formation could be due to the presence of melatonin during the daytime, when it is not normally found in large quantities, or due to the altered circadian rhythm of plasma melatonin. Melatonin stimulates osteoblastic production of Type I Collagen [10], a major component of the organic matrix of cortical bone. Collagen's elastic properties can make bone less brittle, allowing it to sustain greater force without fracturing. Melatonin may alter the arrangement of the collagen matrix in bone, thereby increasing its capacity to resist fracturing. The amount of organic material in bone did not increase in melatonin treated birds, which may indicate that while melatonin does not affect the amount of collagen in developing bone, it could affect properties of the collagen matrix itself by affecting collagen fiber length or patterning.

Birds in this study were treated with melatonin beginning a few days after hatching, continuing throughout skeletal growth and development, and through most of an egg laying cycle. Thus, the changes in bone properties we observed are likely to include developmental changes rather than being restricted to the maintenance of well-developed, mature bone. Bone development consists of primary ossification at the diaphysis, which strengthens cortical bone, and secondary ossification at the epiphyses, which lengthens the bone. Both tibia length and cortical thickness increased in tibias of birds treated with high levels of melatonin, suggesting that melatonin influences both primary bone development and secondary development. Melatonin's influence on both primary and secondary ossification likely yields other changes in bone reorganization which may affect overall strength, but which we were unable to measure.

Each day medullary bone is partially dissolved by osteoclastic activity in order to provide calcium for use in the matrix of the developing egg shell. Our data show an effect of melatonin treatment on the mineral content of medullary bone. Medullary bone is not generally considered to be structural in nature, but can contribute to fracture resistance of bones it occupies [30], [31]. An increase in the amount of medullary bone may improve bone structure's strength by filling the internal cavity in a process known as “sandwich technology” similar to that employed to increase the tensile strength of airframe structures.

Total egg mass was greater in birds treated with melatonin. Melatonin has been shown to delay clutch initiation [32], but not affect the quantity of eggs produced in a laying cycle. Melatonin is found in Japanese quail egg yolks and albumen and has been proposed as part of the antioxidative system that protects the embryo from oxidative stress during development [33]. To our knowledge, the effects of melatonin administration on egg properties have not been elucidated.

Conversely, shell mass of eggs laid by control birds was greater than that of eggs laid by birds treated with melatonin. This suggests that calcium may be preferentially stored in medullary bone or, more likely, melatonin may inhibit the daily resorption of medullary bone by osteoclasts for use in mineralizing egg shells, rendering calcium stores in medullary bone less labile [13]. It is likely that melatonin receptors in the shell gland [6], [9] also play a role in limiting egg shell calcium deposition, likely through epithelial calcium transport. While the bone-strengthening effects of melatonin supplementation in hens could be beneficial for poultry farming, weakened egg shells are not desirable. Melatonin supplementation may be better-suited for birds raised for meat rather than for birds raised for egg production.

In all birds the dietary intake of calcium was equivalent, however melatonin-treated birds deposited less calcium in egg shells than controls, as evidenced by reduced shell weight, while simultaneously affecting an equivalent amount of mineralization in cortical bone. Because hydroxyapatite in bone requires the presence of phosphate, and the calcium carbonate of the egg shell does not, it is possible that phosphate restriction could prevent additional bone mineralization even though calcium is available. Melatonin may affect calcium absorption, as previously reported [16], but a failure to absorb a proportionate amount of phosphorus could explain the unchanged bone mineral content in melatonin-treated birds. If this is not the case, excess calcium may be excreted if it is not used to ossify bone or mineralize egg shells.

Overall, our findings demonstrate that chronic melatonin treatment increases leg bone strength and bone length in laying hens. It is likely that these properties are influenced by melatonin during early development. Conversely, the effects of melatonin on egg shells are likely the result of melatonin's effects on calcium transport within the shell gland. Future studies examining the effects of melatonin directly on the function of the shell gland and medullary bone in vitro would be helpful in elucidating how these processes.

## Supporting Information

Table S1 **Upper yield point, yield strength, break force, and elastic force of keels collected from White Leghorn hens after melatonin treatment.**
(DOCX)
