# Supplementary material for: The Effects of Melatonin on the Physical Properties of Bones and Egg Shells in the Laying Hen
Source: PLoS One. 2013 Feb 28;8(2):e55663. doi: 10.1371/journal.pone.0055663 (PMC3585294; doi:10.1371/journal.pone.0055663)
Supplement: Table S1 — Upper yield point, yield strength, break force, and elastic force of keels collected from White Leghorn hens after melatonin treatment. (DOCX) [file pone.0055663.s001.docx]

Table S1: Keel Strength Properties

| Group | Upper yield point (N) | Yield strength (N) | Breaking force (N) | Elastic force (N) |
| --- | --- | --- | --- | --- |
| Control | 27.31 (±5.55) | 41.88 (±7.90) | 68.80 (±10.77) | 411509.08 (±108963.30) |
| Low | 13.44 (±0.67) | 19.29 (±0.98) | 111.73 (±29.53) | 43938.72 (±53136.18) |
| Medium | 22.74 (±5.17) | 37.11 (±4.36) | 75.29 (±16.25) | 234314.84 (±83048.17) |
| High | 25.62 (±4.79) | 35.10 (±4.44) | 70.19 (±10.65) | 463251.2 (±126932.23) |

^*^ = significant at p<0.05

^**^ = significant at P<0.01

^***^ = significant at p<0.001

Data given are mean ± SE.

Table S1: Upper yield point, yield strength, break force, and elastic force of keels collected from White Leghorn hens after melatonin treatment.
